# Supplementary material for: The Distribution of Circulating Tumor Cells Is Different in Metastatic Lobular Compared to Ductal Carcinoma of the Breast—Long-Term Prognostic Significance
Source: Cells. 2020 Jul 17;9(7):1718. doi: 10.3390/cells9071718 (PMC7407940; doi:10.3390/cells9071718)
Supplement: Supplementary file 1 [file cells-09-01718-s001.zip › suppl Table S1.docx]

**Supplementary material 2**

CTC and CA15-3 distributions

| **CTC status** | | **BL** | **1M** | **3M** | **6M** |
| --- | --- | --- | --- | --- | --- |
| **CTC**  **Median (Range)** | ILC | 70 (0-2598) | 4 (0-85) | 2 (0-181) | 1 (0-184) |
|  | NST | 2 (0-668) | 0 (0-263) | 0 (0-144) | 0 (0-695) |
| **CTC ≥5** | ILC | 22/28 (79%) | 13/27 (48%) | 7/27 (26%) | 7/26 (27%) |
|  | NST | 49/107 (46%) | 24/94 (26%) | 13/80 (16%) | 5/65 (8%) |
| **CTC ≥20** | ILC | 17/28 (61%) | 6/27 (22%) | 3/27 (11%) | 3/26 (12%) |
|  | NST | 32/107 (30%) | 19/94 (20%) | 8/80 (10%) | 4/65 (6%) |
| **CTC ≥80** | ILC | 12/28 (43%) | 1/27 (4%) | 1/27 (4%) | 2/26 (8%) |
|  | NST | 16/107(15%) | 5/94 (5%) | 2/80 (3%) | 3/65 (5%) |
| **CA15-3 status** | |  |  |  |  |
| **CA15-3**  **Median** (Range) | ILC | 392 (17-2999) | 346 (20-2761) | 169 (20-703) | 89 (16-943) |
|  | NST | 91 (6-2999) | 61 (6-1972) | 56 (7-1911) | 42 (7-700) |
| **CA15-3 ≥30** | ILC | 23/27 (85%) | 21/26 (81%) | 19/25 (76%) | 20/25 (80%) |
|  | NST | 79/104 (76%) | 68/91 (75%) | 55/78 (71%) | 39/62 (63%) |
| **CA15-3 ≥200** | ILC | 16/27 (59%) | 16/26 (61%) | 9/25 (36%) | 6/25 (24%) |
|  | NST | 31/104 (30%) | 24/91 (26%) | 13/78 (17%) | 6/62 (10%) |
| **CA15-3 ≥400** | ILC | 13/27 (48%) | 12/26 (46%) | 6/25 (24%) | 3/25 (12%) |
|  | NST | 20/104 (19%) | 20/91 (22%) | 9/78 (12%) | 2/62 (3%) |
