# Supplementary material for: The Distribution of Circulating Tumor Cells Is Different in Metastatic Lobular Compared to Ductal Carcinoma of the Breast—Long-Term Prognostic Significance
Source: Cells. 2020 Jul 17;9(7):1718. doi: 10.3390/cells9071718 (PMC7407940; doi:10.3390/cells9071718)
Supplement: Supplementary file 1 [file cells-09-01718-s001.zip › suppl Figure S3.pdf]

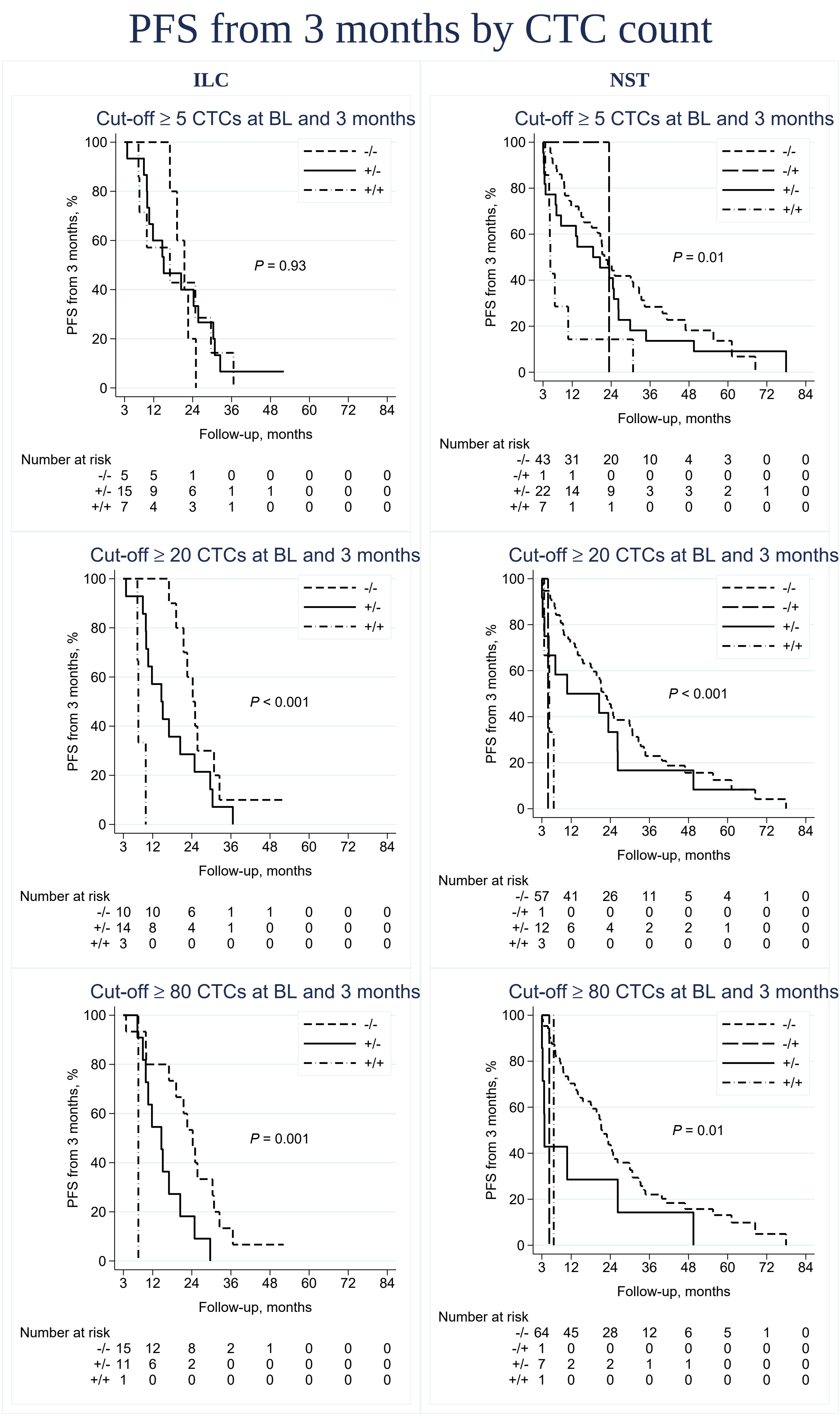
PFS from 3 months by CTC count
ILC
NST
Cut-off ≥ 5 CTCs at BL and 3 months
-/-
+/-
+/+
P = 0.93
PFS from 3 months, %
Follow-up, months
Number at risk
-/- 5 5 1 0 0 0 0 0
+/- 15 9 6 1 1 0 0 0
+/+ 7 4 3 1 0 0 0 0
Cut-off ≥ 5 CTCs at BL and 3 months
-/-
-/+
+/-
+/+
P = 0.01
PFS from 3 months, %
Follow-up, months
Number at risk
-/- 43 31 20 10 4 3 0 0
-/+ 1 1 0 0 0 0 0 0
+/- 22 14 9 3 3 2 1 0
+/+ 7 1 1 0 0 0 0 0
Cut-off ≥ 20 CTCs at BL and 3 months
-/-
+/-
+/+
P < 0.001
PFS from 3 months, %
Follow-up, months
Number at risk
-/- 10 10 6 1 1 0 0 0
+/- 14 8 4 1 0 0 0 0
+/+ 3 0 0 0 0 0 0 0
Cut-off ≥ 20 CTCs at BL and 3 months
-/-
-/+
+/-
+/+
P < 0.001
PFS from 3 months, %
Follow-up, months
Number at risk
-/- 57 41 26 11 5 4 1 0
-/+ 1 0 0 0 0 0 0 0
+/- 12 6 4 2 2 1 0 0
+/+ 3 0 0 0 0 0 0 0
Cut-off ≥ 80 CTCs at BL and 3 months
-/-
+/-
+/+
P = 0.001
PFS from 3 months, %
Follow-up, months
Number at risk
-/- 15 12 8 2 1 0 0 0
+/- 11 6 2 0 0 0 0 0
+/+ 1 0 0 0 0 0 0 0
Cut-off ≥ 80 CTCs at BL and 3 months
-/-
-/+
+/-
+/+
P = 0.01
PFS from 3 months, %
Follow-up, months
Number at risk
-/- 64 45 28 12 6 5 1 0
-/+ 1 0 0 0 0 0 0 0
+/- 7 2 2 1 1 0 0 0
+/+ 1 0 0 0 0 0 0 0
