# Supplementary material for: The Distribution of Circulating Tumor Cells Is Different in Metastatic Lobular Compared to Ductal Carcinoma of the Breast—Long-Term Prognostic Significance
Source: Cells. 2020 Jul 17;9(7):1718. doi: 10.3390/cells9071718 (PMC7407940; doi:10.3390/cells9071718)

OS from 3 months by CTC count

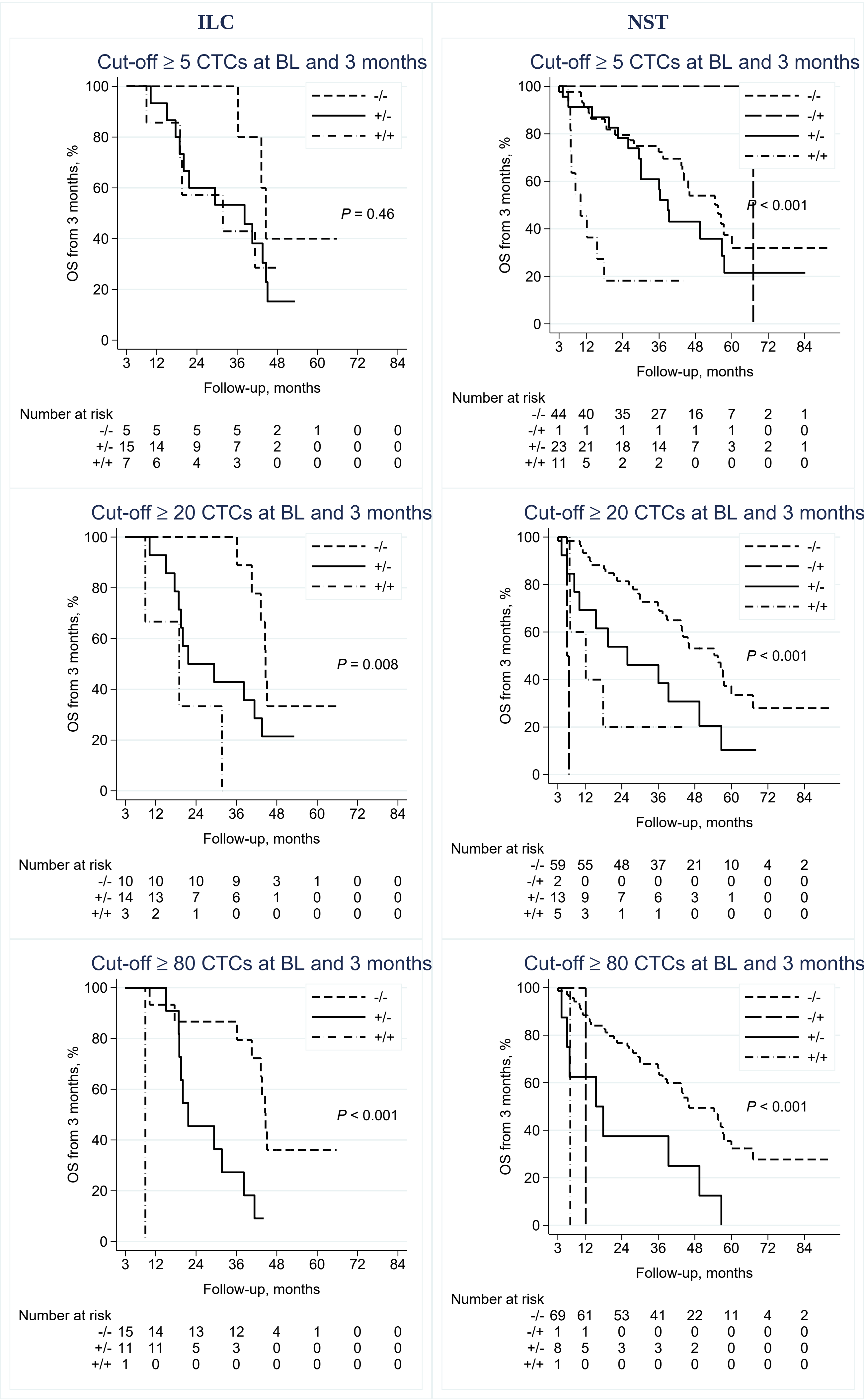

Supplement: Supplementary file 1 [file cells-09-01718-s001.zip › suppl Figure S4.pdf]
