# Supplementary material for: The Distribution of Circulating Tumor Cells Is Different in Metastatic Lobular Compared to Ductal Carcinoma of the Breast—Long-Term Prognostic Significance
Source: Cells. 2020 Jul 17;9(7):1718. doi: 10.3390/cells9071718 (PMC7407940; doi:10.3390/cells9071718)

Supplementary material 7

Progression-free survival (PFS) by CA 15-3 cut-off ≥30 in invasive lobular carcinoma (ILC) (*P*=0.91)

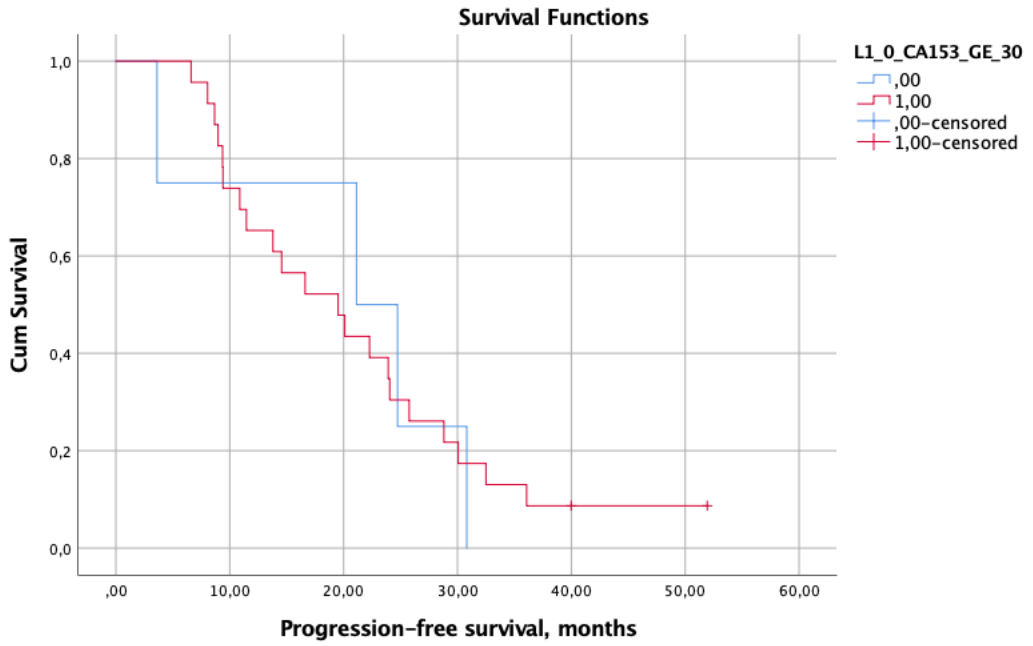

Overall survival (OS) by CA 15-3 cut-off ≥30 in ILC (*P*=0.28)

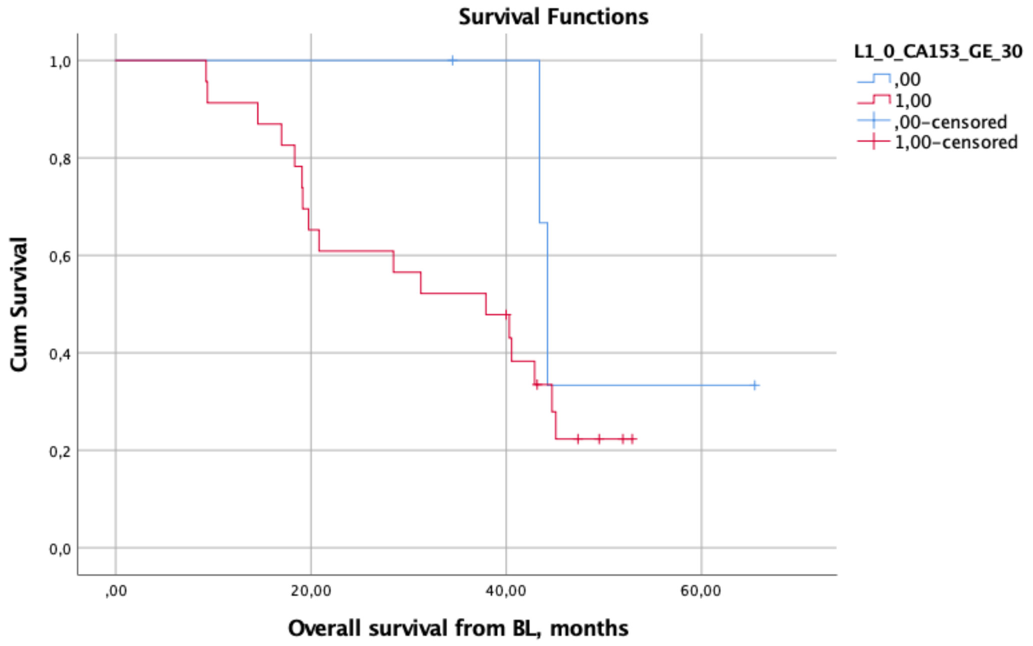

PFS by CA 15-3 cut-off ≥100 in ILC (*P*=0.46)

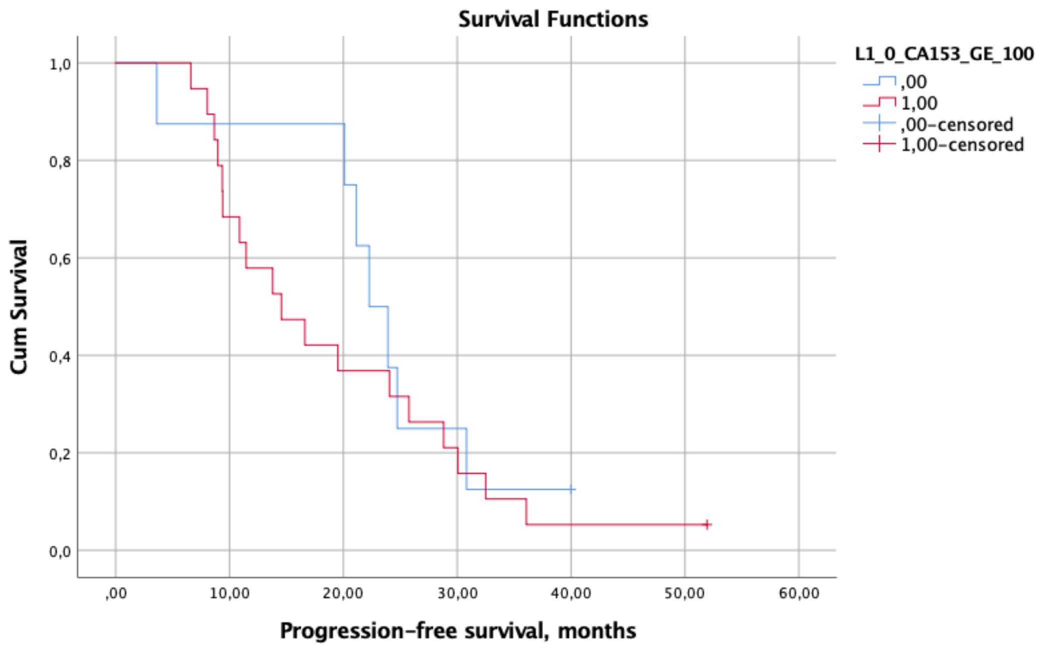

OS by CA 15-3 cut-off ≥100 in ILC (*P*=0.03)

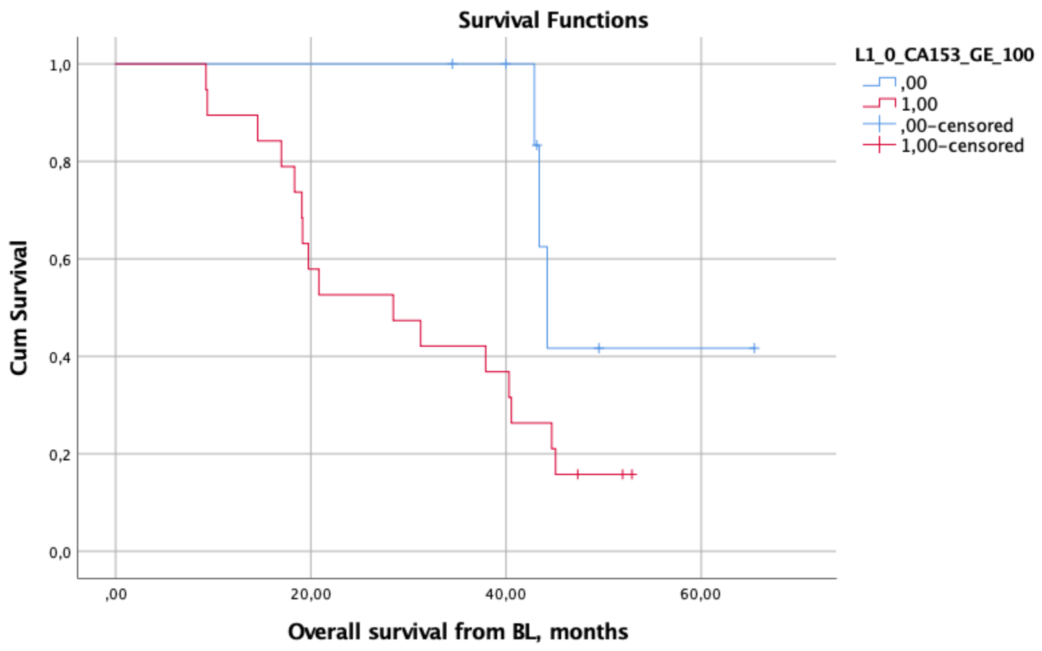

PFS by CA 15-3 cut-off ≥200 in ILC (P=0.44)

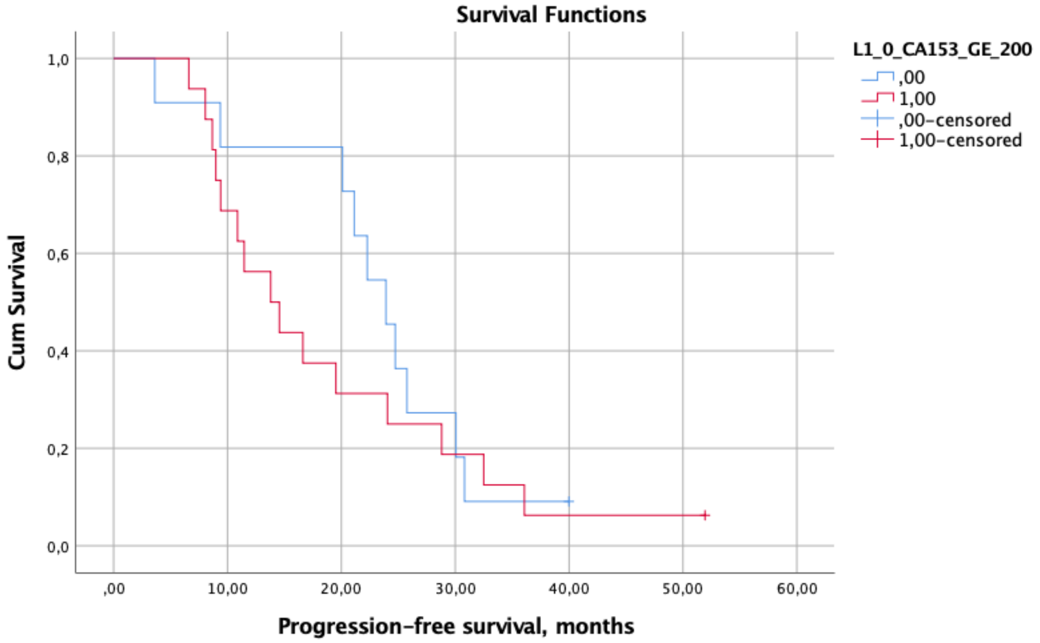

OS by CA 15-3 cut-off ≥200 in ILC (P=0.01)

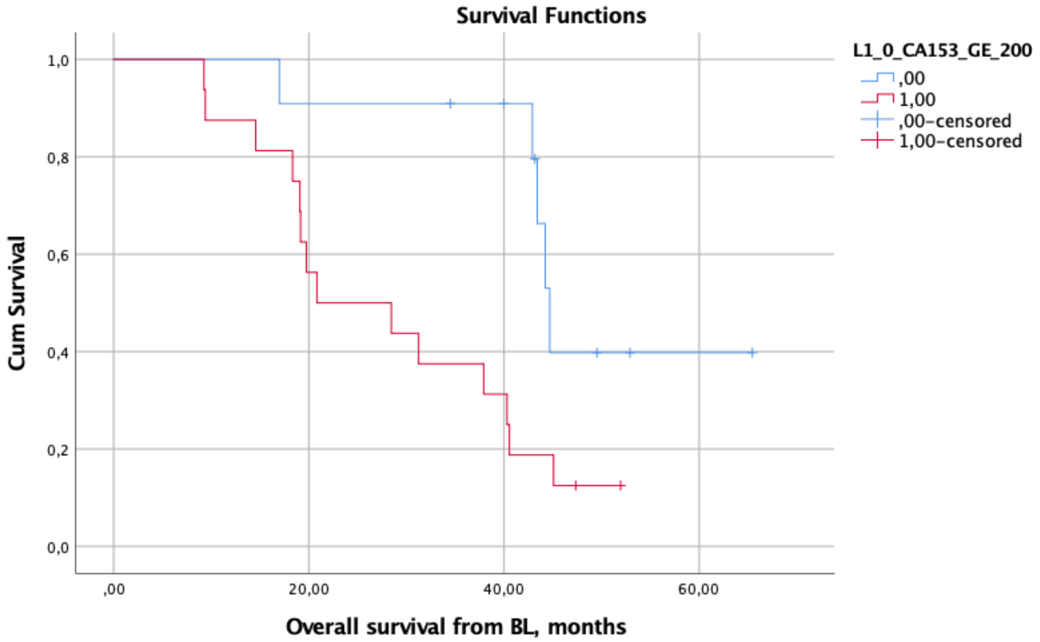

PFS by CA 15-3 cut-off ≥400 in ILC (P=0.27)

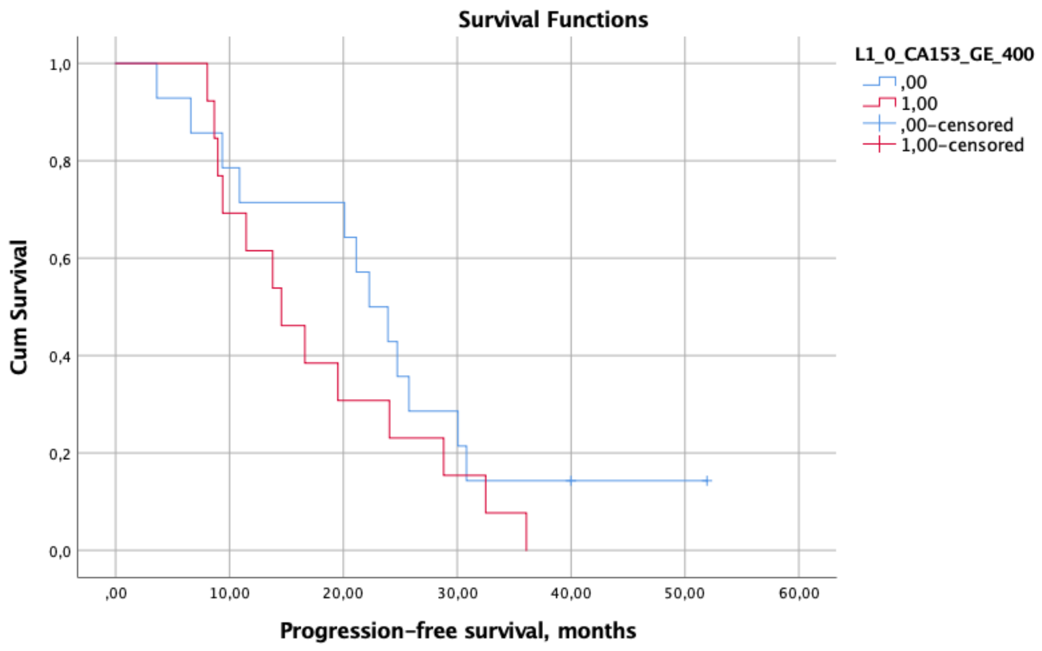

OS by CA 15-3 cut-off ≥400 in ILC (P=0.01)

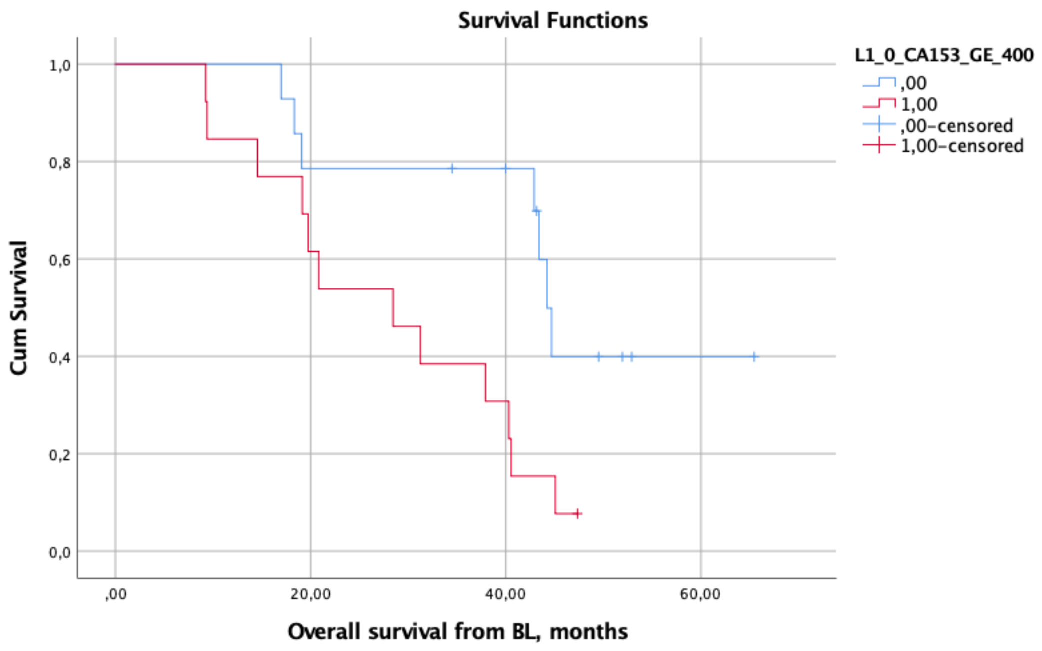

PFS by CA15-3 cut-off ≥30 in invasive ductal carcinoma of no special type (NST) (P=0.26)

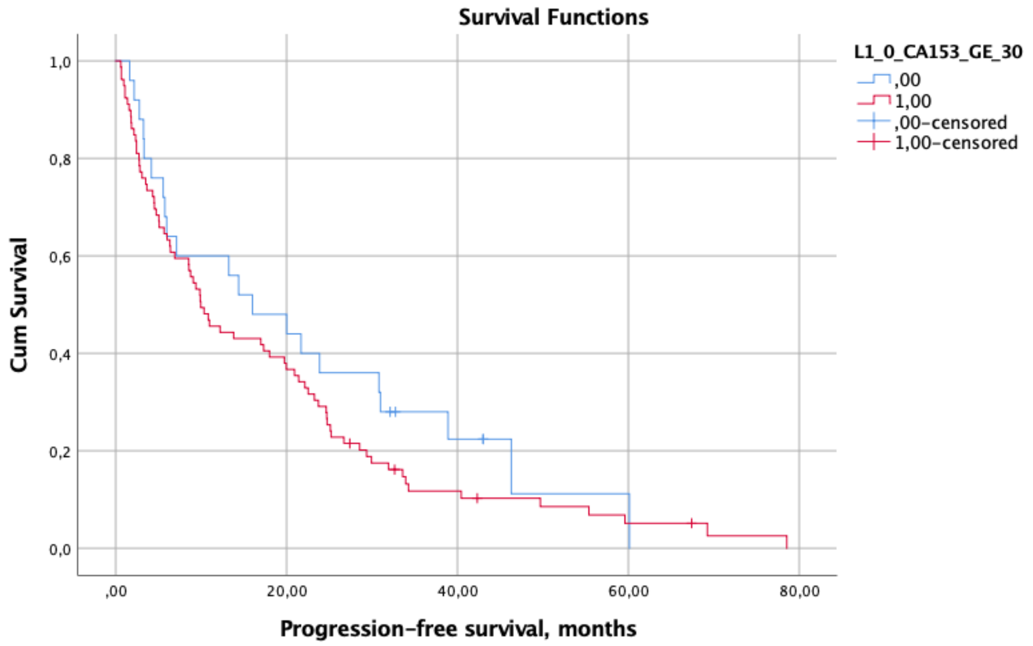

OS by CA 15-3 cut-off ≥30 in NST (P=0.14)

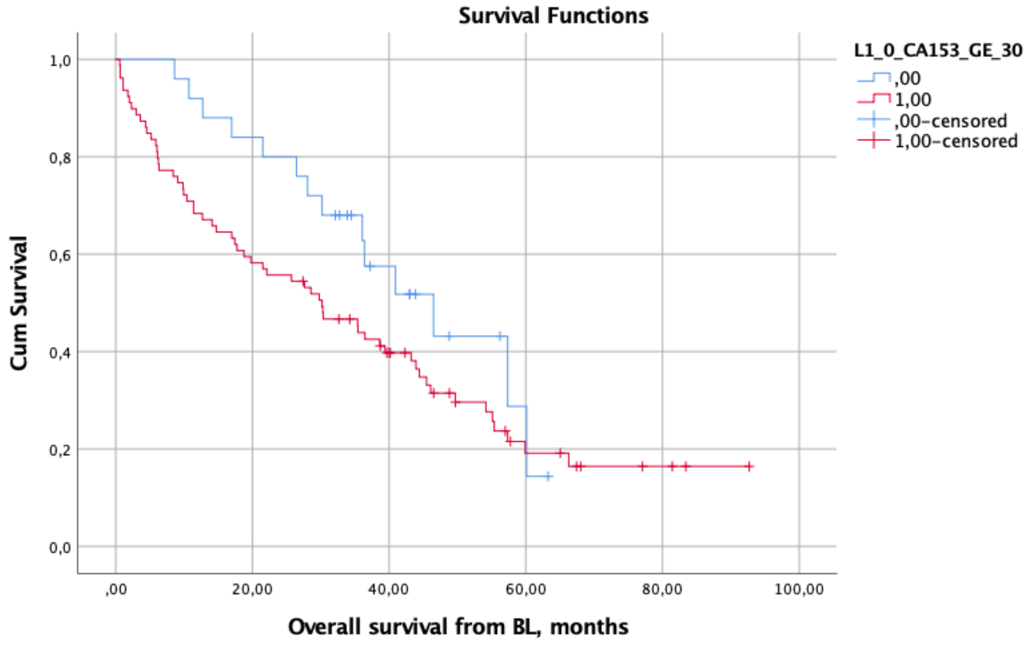

PFS by CA 15-3 cut-off ≥100 in NST (*P*=0.03)

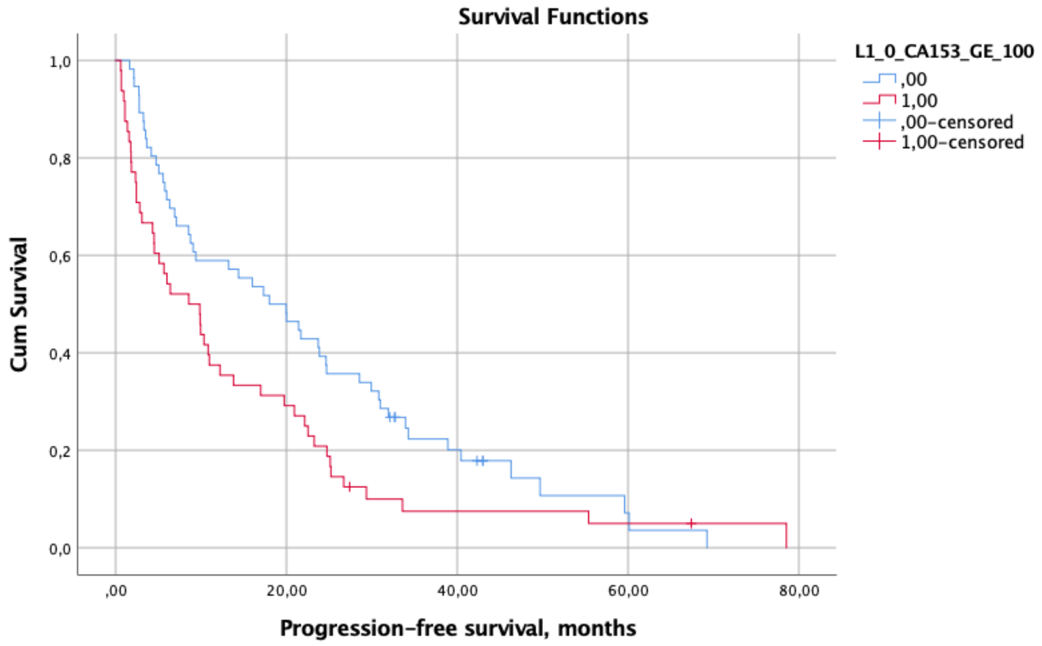

OS by CA 15-3 cut-off ≥100 in NST (*P*<0.001)

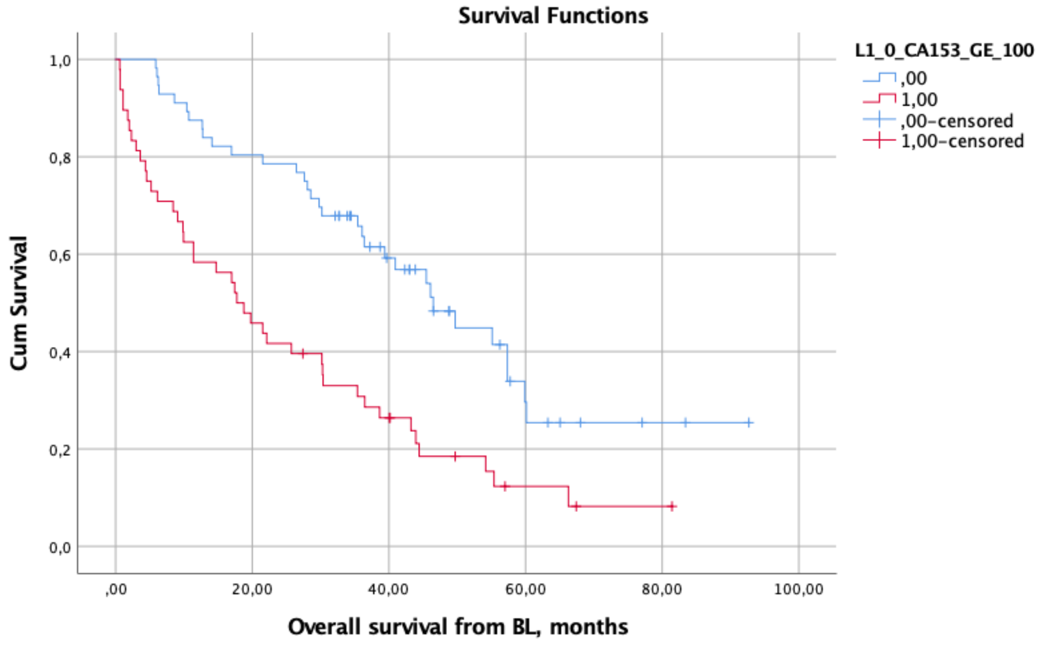

PFS by CA 15-3 cut-off ≥200 in NST (*P*=0.35)

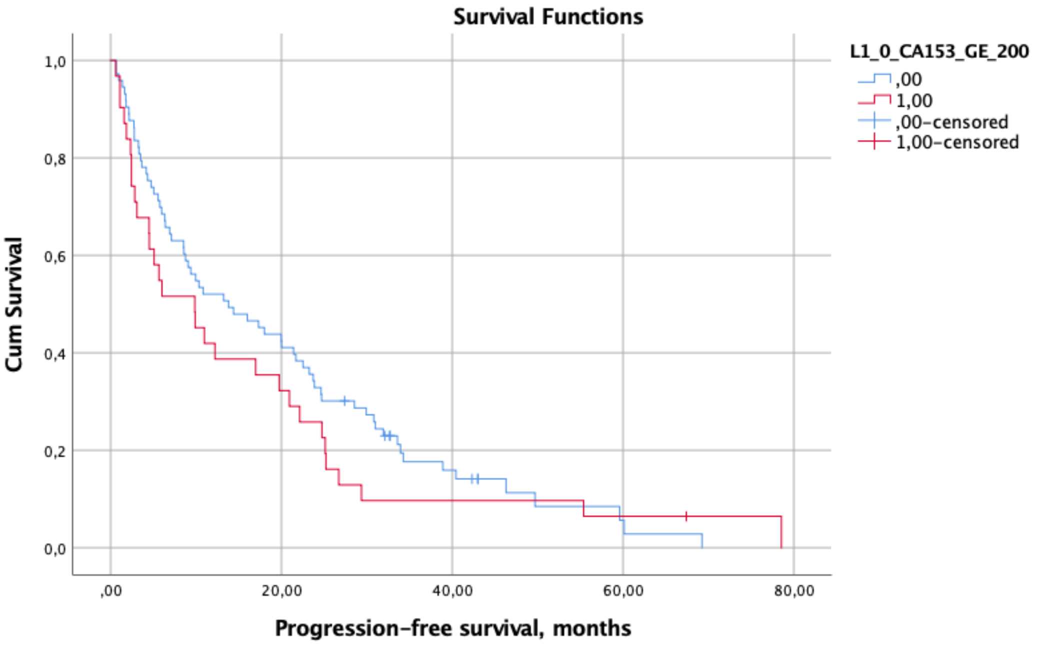

OS by CA 15-3 cut-off ≥200 in NST (*P*=0.01)

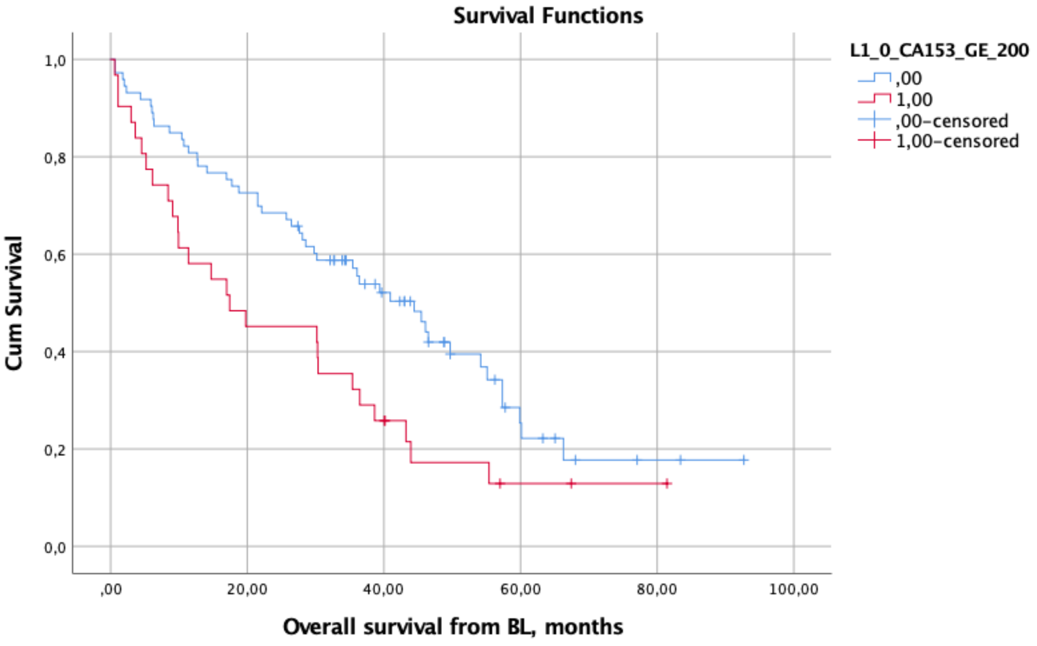

PFS by CA 15-3 cut-off ≥400 in NST (*P*=0.22)

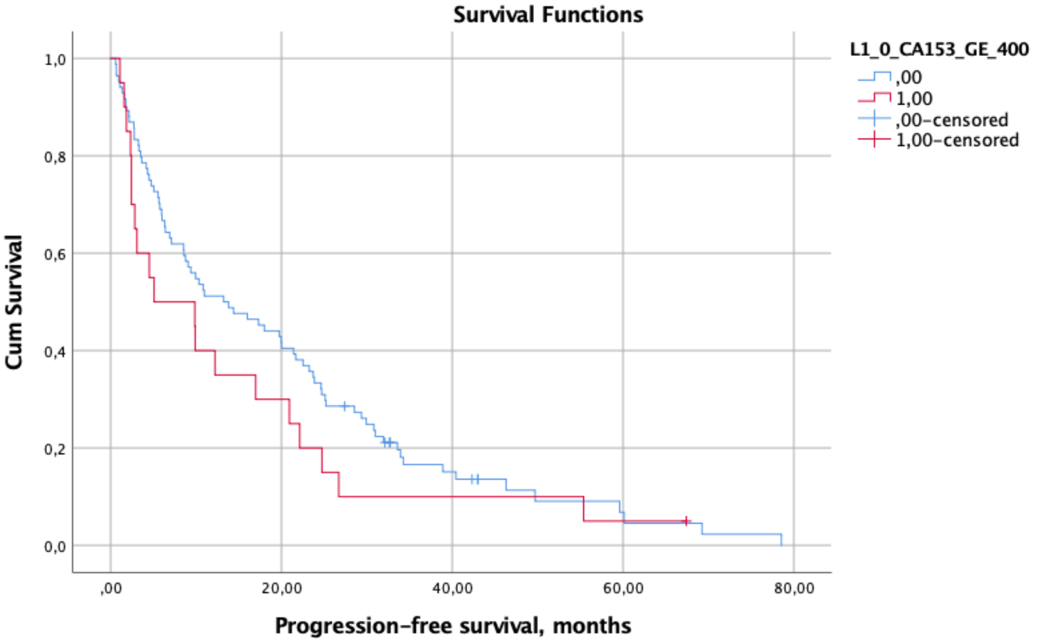

OS by CA 15-3 cut-off ≥400 in NST (*P*=0.01)

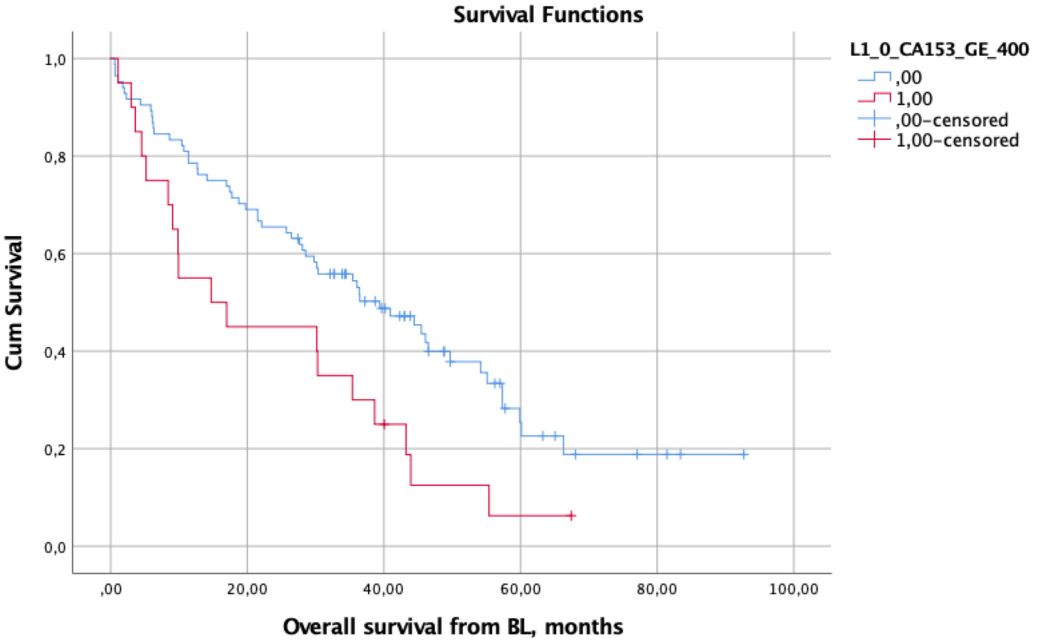

Supplement: Supplementary file 1 [file cells-09-01718-s001.zip › suppl Figure S7.pdf]
