# Supplementary figures and images for: The Distribution of Circulating Tumor Cells Is Different in Metastatic Lobular Compared to Ductal Carcinoma of the Breast—Long-Term Prognostic Significance
Source: Cells. 2020 Jul 17;9(7):1718. doi: 10.3390/cells9071718 (PMC7407940; doi:10.3390/cells9071718)

# Survival by histopathological type

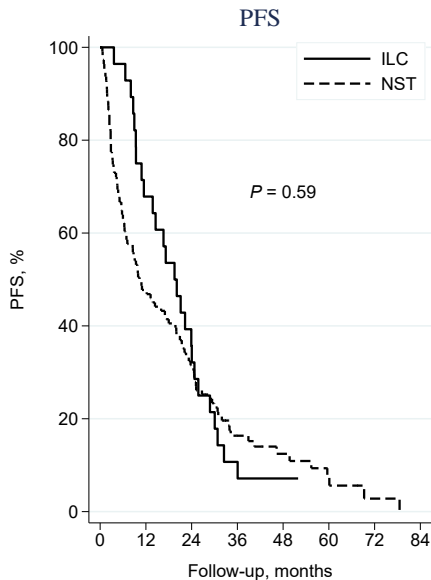

Number at risk

|     |     |    |    |    |   |   |   |   |
|-----|-----|----|----|----|---|---|---|---|
| ILC | 28  | 19 | 10 | 3  | 1 | 0 | 0 | 0 |
| NST | 111 | 53 | 34 | 14 | 8 | 4 | 1 | 0 |

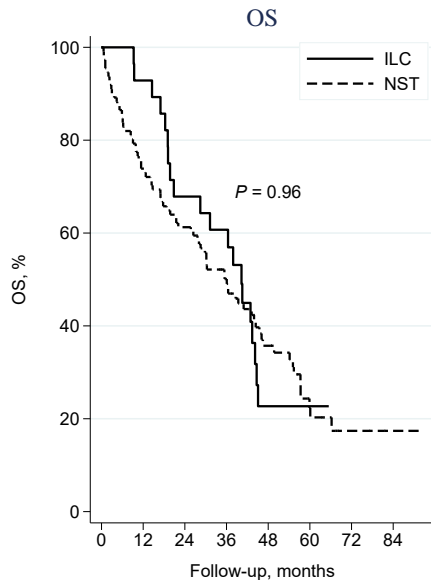

Number at risk

|     |     |    |    |    |    |    |   |   |
|-----|-----|----|----|----|----|----|---|---|
| ILC | 28  | 26 | 19 | 16 | 4  | 1  | 0 | 0 |
| NST | 111 | 82 | 68 | 48 | 26 | 11 | 4 | 1 |

Supplement: Supplementary file 1 [file cells-09-01718-s001.zip › suppl Figure S2.pdf]

# PFS by CTC Cluster

## ILC

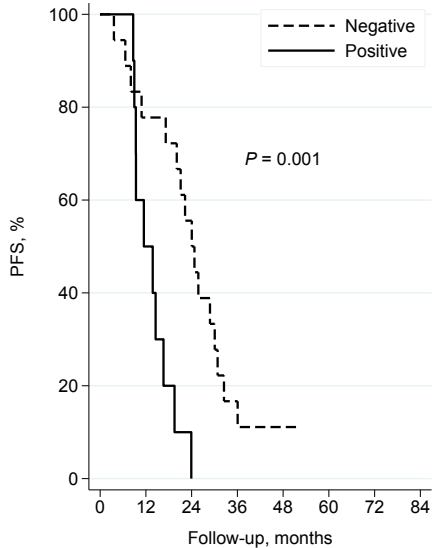

|                |    |    |    |   |   |   |   |   |
|----------------|----|----|----|---|---|---|---|---|
| Number at risk |    |    |    |   |   |   |   |   |
| Negative       | 18 | 14 | 10 | 3 | 1 | 0 | 0 | 0 |
| Positive       | 10 | 5  | 0  | 0 | 0 | 0 | 0 | 0 |

## NST

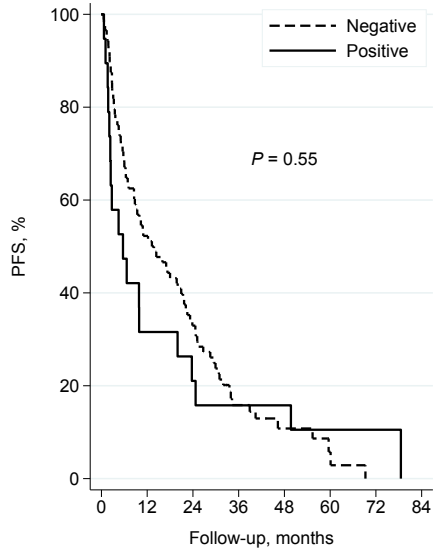

|                |    |    |    |    |   |   |   |   |
|----------------|----|----|----|----|---|---|---|---|
| Number at risk |    |    |    |    |   |   |   |   |
| Negative       | 88 | 46 | 29 | 11 | 5 | 2 | 0 | 0 |
| Positive       | 19 | 6  | 4  | 3  | 3 | 2 | 1 | 0 |

Supplement: Supplementary file 1 [file cells-09-01718-s001.zip › suppl Figure S5.pdf]

# OS by CTC Cluster

## ILC

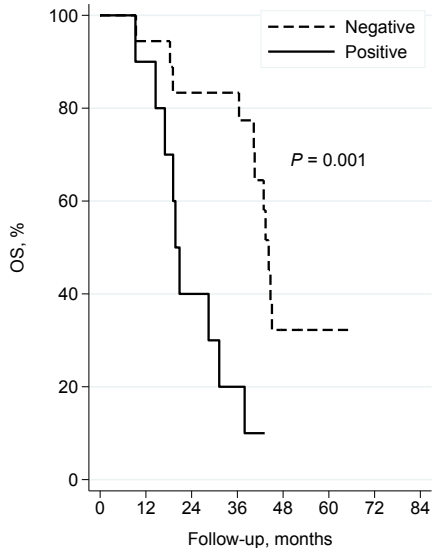

|                |    |    |    |    |   |   |   |   |
|----------------|----|----|----|----|---|---|---|---|
| Number at risk |    |    |    |    |   |   |   |   |
| Negative       | 18 | 17 | 15 | 14 | 4 | 1 | 0 | 0 |
| Positive       | 10 | 9  | 4  | 2  | 0 | 0 | 0 | 0 |

## NST

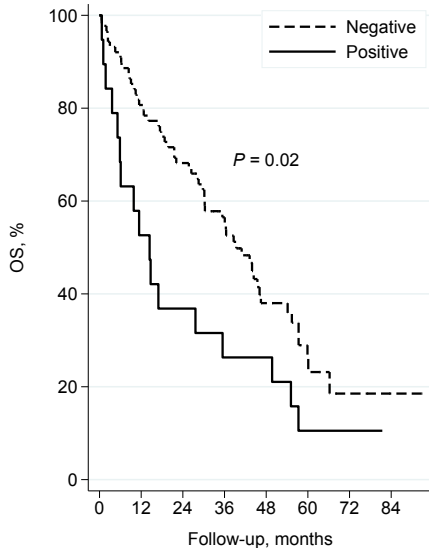

|                |    |    |    |    |    |   |   |   |
|----------------|----|----|----|----|----|---|---|---|
| Number at risk |    |    |    |    |    |   |   |   |
| Negative       | 88 | 71 | 60 | 43 | 21 | 9 | 3 | 1 |
| Positive       | 19 | 10 | 7  | 5  | 5  | 2 | 1 | 0 |

Supplement: Supplementary file 1 [file cells-09-01718-s001.zip › suppl Figure S6.pdf]

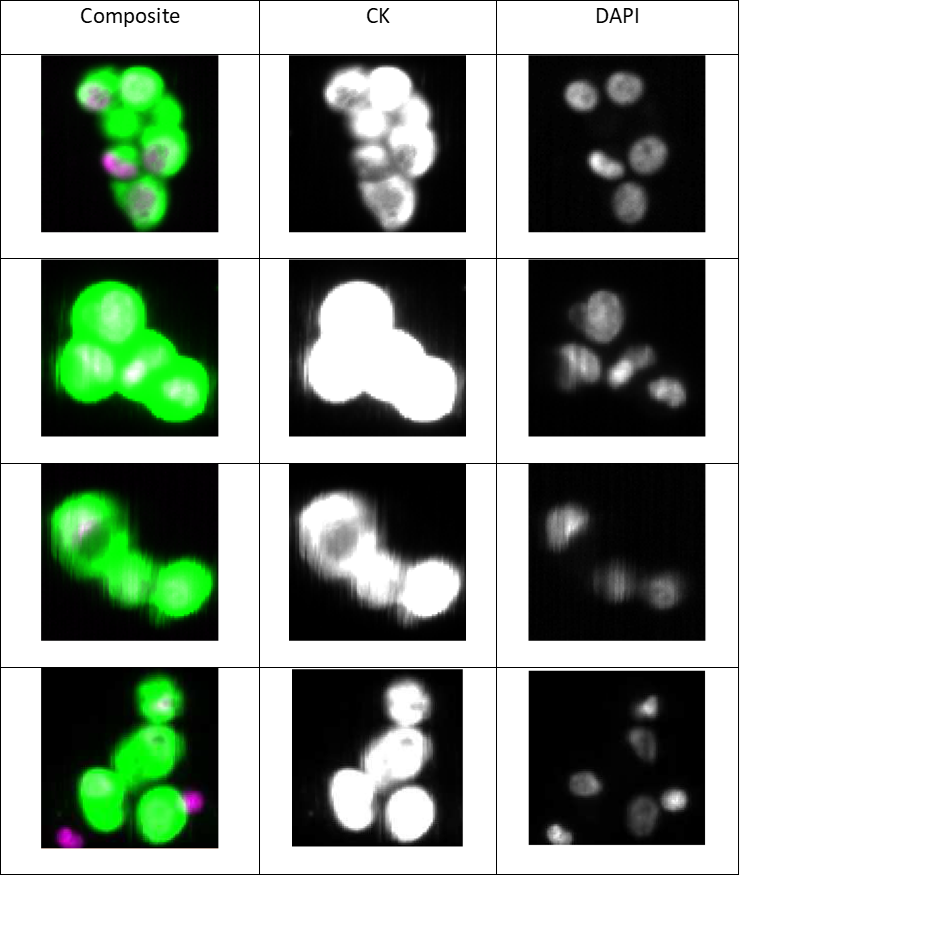

Supplement: Supplementary file 1 [file cells-09-01718-s001.zip › suppl Figure S1.tif]
